# Supplementary material for: Distribution of DC Subtypes: CD83+, DC-LAMP+, CD1a+, CD1c+, CD123+, and DC-SIGN+ in the Tumor Microenvironment of Endometrial Cancers—Correlation with Clinicopathologic Features
Source: Int J Mol Sci. 2023 Jan 18;24(3):1933. doi: 10.3390/ijms24031933 (PMC9915342; doi:10.3390/ijms24031933)
Supplement: Supplementary file 1 [file ijms-24-01933-s001.zip › ijms-2097461-supplementary.pdf]

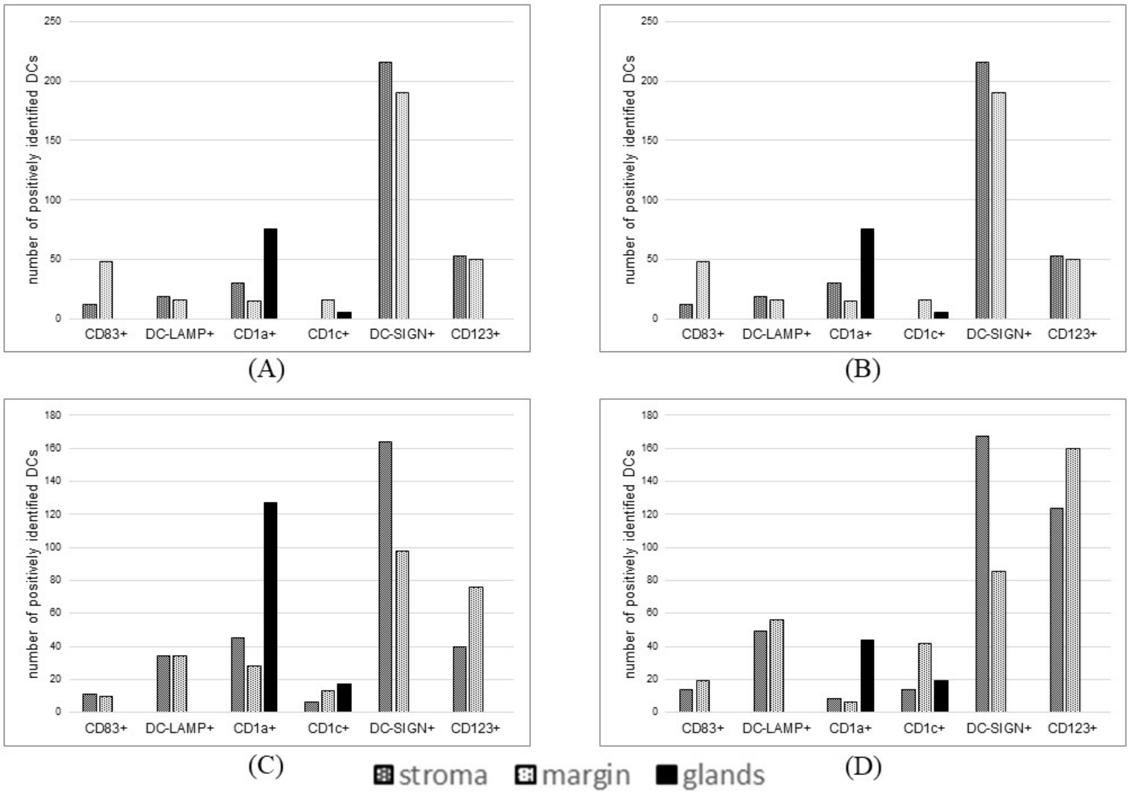

Figure S1. Counts [/1mm2] of DCs positively identified for the stated antibodies, in different ar-eas (stroma, margin, glands) of endometrial neoplasms: **(A)** clear cell carcinoma; **(B)** mucinous carcinoma; **(C)** mixed carcinoma; **(D)** carcinosarcoma.

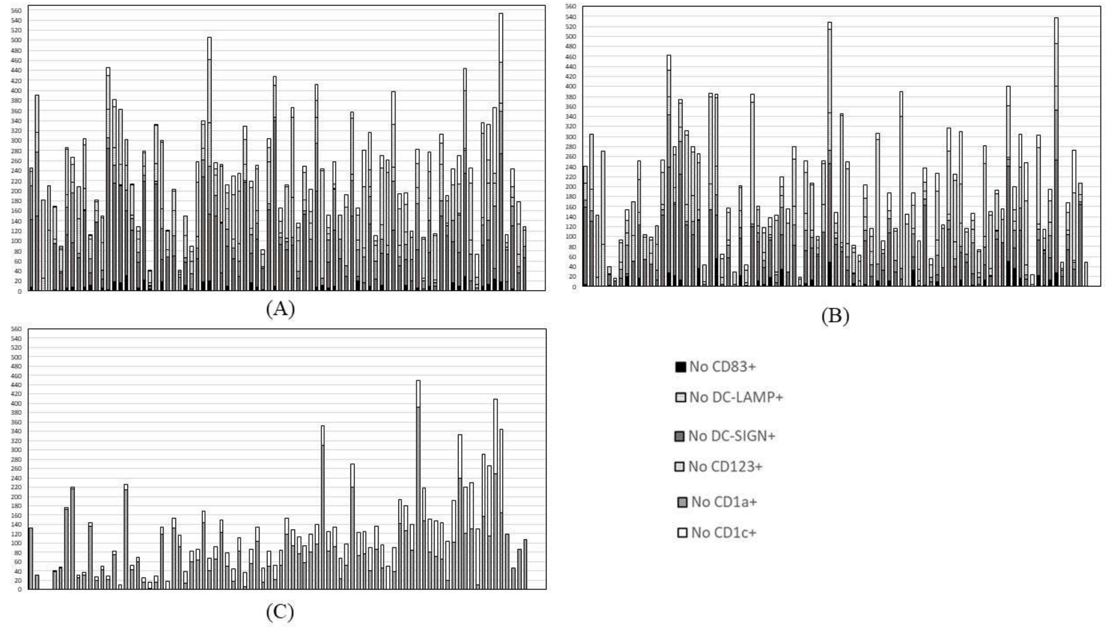

Figure S2. Counts [/1mm2] of DCs positively identified for the stated antibodies, presented for every sample individually, in different areas of cancerous tissues: **(A)** tumor stroma; **(B)** invasive margin; **(C)** tumor glands (CD1a+ and CD1c+ DCs were the only DCs subtypes found in glandular epithelium).

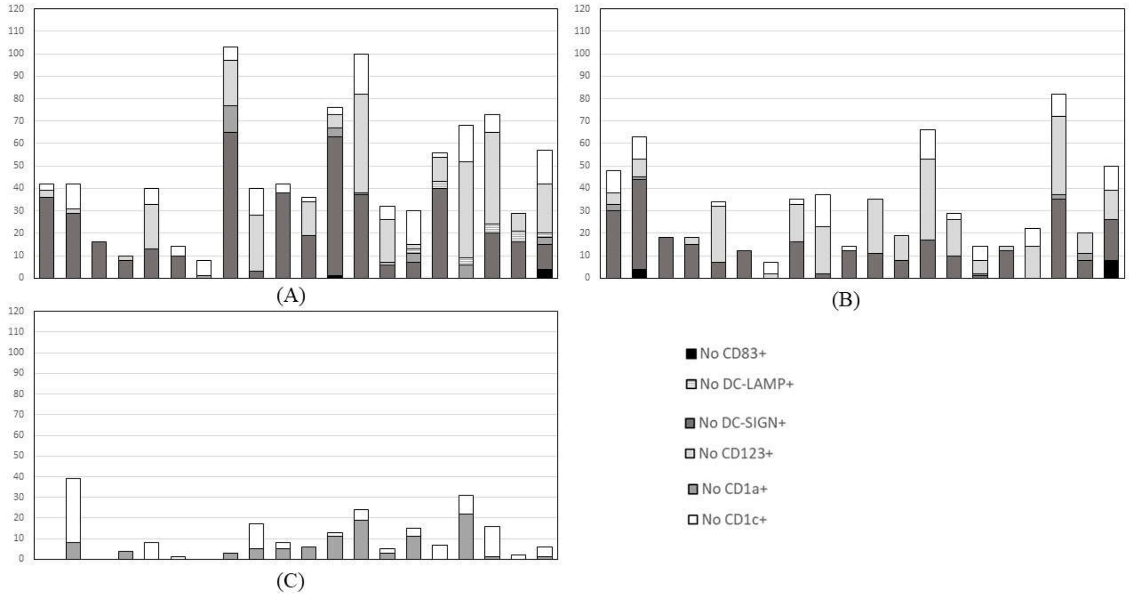

Figure S3. Counts [/1mm2] of DCs positively identified for the stated antibodies, presented for every sample individually, in **(A)** normal endometrial stroma; **(B)** normal endometrial margin; **(C)** normal endometrial glands (CD1a+ and CD1c+ DCs were the only DCs subtypes found in nor-mal glandular epithelium). Areas of non-cancerous endometrium within ECs' slides were initially examined at 10× magnification; within areas most abundant in positively identified DCs, their number from five representative fields of view was summed up at high 40× magnification and expressed per 1 mm2.

Table S2. Associations between counts of different DCs subtypes and clinicopathological fea-tures of endometrioid cancers (ECs). For every DCs subtype, compared cancer groups were dis-tinguished based on either histological grade (3 groups: grade 1, 2, 3), pT score (six groups: pT1, pT1a, pT1b, pT2, pT3a, pT3b), or pN score (3 groups: pN0, pN1, pN2). The numbers of samples allocated to different grade or stage scores are unevenly distributed between different antibodies subgroups, increasing the risk of biased results. The statistics were calculated using the Krustal-Wallis ANOVA; * $p < 0.05$ was considered statistically significant
pT, pN – elements of pathological TNM classification established for uterine cancers

Table S2. Associations between counts of different DCs subtypes and clinicopathological features of ECs

dependent variable	independent (grouping) variable	p-value *
DC CD83+ stroma	Grade (3 groups)	0.7468
DC DC-SIGN+ stroma		0.9557
DC CD1a+ stroma		0.3100
DC DC-LAMP+ stroma		0.0540
DC CD123+ stroma		0.4190
DC CD1c+ stroma		0.1138
DC CD83+ margin		0.6624
DC DC-SIGN+ margin		0.5754
DC CD1a+ margin		0.1879
DC DC-LAMP+ margin		0.0897
DC CD123+ margin		0.4359

DC CD1c+ margin		0.4471
DC CD1a+ glands		0.6203
DC CD1c+ glands		0.0977
DC CD83+ stroma	Stage: pT (6 groups)	0.2054
DC DC-SIGN+ stroma		0.8625
DC CD1a+ stroma		0.6990
DC DC-LAMP+ stroma		0.7274
DC CD123+ stroma		0.7943
DC CD1c+ stroma		0.3045
DC CD83+ margin		0.1066
DC DC-SIGN+ margin		0.8745
DC CD1a+ margin		0.4552
DC DC-LAMP+ margin		0.7943
DC CD123+ margin		0.2407
DC CD1c+ margin		0.7928
DC CD1a+ glands		0. 3568
DC CD1c+ glands		0.4303
DC CD83+ stroma	Stage: pN (3 groups)	0.6773
DC DC-SIGN+ stroma		0.2926
DC CD1a+ stroma		0.2545
DC DC-LAMP+ stroma		0.5580
DC CD123+ stroma		0.5853
DC CD1c+ stroma		0.9522
DC CD83+ margin		0.4261
DC DC-SIGN+ margin		0.6166
DC CD1a+ margin		0.3360
DC DC-LAMP+ margin		0.8122
DC CD123+ margin		0.5893
DC CD1c+ margin		0.4168
DC CD1a+ glands		0.3053
DC CD1c+ glands		0.4110
